# Anti-Mitochondrial Antibody Titers Decrease Over Time in Primary Biliary Cholangitis Patients With Ursodeoxycholic Acid Therapeutic Response: A Cohort Study Followed Up to 28 Years

**DOI:** 10.3389/fimmu.2022.869018

**Published:** 2022-05-19

**Authors:** Ming-Ling Chang, Wei-Ting Chen, Tien-Ming Chan, Cheng-Yu Lin, Ming-Yu Chang, Shiang-Chi Chen, Rong-Nan Chien

**Affiliations:** ^1^ Division of Hepatology, Department of Gastroenterology and Hepatology, Chang Gung Memorial Hospital, Taoyuan, Taiwan; ^2^ Department of Medicine, College of Medicine, Chang Gung University, Taoyuan, Taiwan; ^3^ Division of Rheumatology, Allergy, and Immunology, Department of Internal Medicine, Chang Gung Memorial Hospital, Taoyuan, Taiwan; ^4^ Division of Pediatrics, Chang Gung Memorial Hospital, Keelung, Taiwan; ^5^ Department of Nursing, Taipei Medical University, Taipei, Taiwan

**Keywords:** antimitochondrial antibodies (AMA), primary biliary cholangitis (PBC), UDCA, alkaline phospatase, cirrhosis

## Abstract

**Background:**

How anti-mitochondrial antibody (AMA) and liver biochemistry levels change in primary biliary cholangitis (PBC) patients treated with ursodeoxycholic acid (UDCA) remains unclear.

**Methods:**

A 28-year cohort of 157 PBC patients was conducted. Patients with alkaline phosphatase (Alk-p) levels >1.67 × upper limit of normal after 1 year of UDCA treatment were considered nonresponders.

**Results:**

At baseline, of 157 (mean age: 54.41 years), 136 (86.6%) were female, 51 (32.5%) had cirrhosis, and 128 (81.5%) had detectable AMAs (immunoglobulin G). UDCA nonresponders (n=61) were younger and had higher Alk-p and total bilirubin levels and cirrhosis rates than UDCA responders (n=84). Alk-p levels and cirrhosis were negatively associated with UDCA response. Regardless of cirrhosis and UDCA response, most PBC patients had decreased Alk-p and γ-glutamyltransferase levels at last follow-up (up to 28.73 years) compared with baseline levels. Patients with baseline cirrhosis (2.78 ± 2.56 vs. 6.84 ± 9.00 mg/dL, *p*=0.024) and UDCA nonresponders (2.54 ± 2.19 vs. 4.51 ± 6.99 mg/dL, *p*=0.006) had increased total bilirubin levels while patients without cirrhosis (AST: 91.5 ± 84.5 vs. 58.9 ± 43.7 U/L, *p*<0.001; ALT: 107.3 ± 122.5 vs. 50.7 ± 36.8 U/L, *p*<0.001) and UDCA responders (AST: 83.8 ± 101.3 vs. 45.58 ± 38.42 U/L, *p*=0.014; ALT: 95.10 ± 144.6 vs. 39.12 ± 30.65 U/L, *p*=0.009) had decreased aminotransferase levels. Only UDCA responders had decreased AMA titers from 1 year after UDCA treatment (*p*=0.028) until the last follow-up (*p*<0.001).

**Conclusions:**

UDCA responders exhibited decreased AMA titers 1 year after treatment. Regardless of UDCA response, PBC patients showed improved cholestatic features, but only UDCA responders and patients without baseline cirrhosis exhibited attenuated hepatobiliary damage following UDCA treatment.

## Introduction

Primary biliary cholangitis (PBC), formerly called primary biliary cirrhosis, is characterized by a triad of chronic cholestasis, circulating anti-mitochondrial antibodies (AMAs), and characteristic liver biopsy findings of nonsuppurative destructive cholangitis and interlobular bile duct destruction (1). PBC is an idiopathic chronic autoimmune liver disease that predominantly affects middle-aged women (2). Most PBC patients eventually develop hepatic fibrosis and may require liver transplantation during the late stage of the disease if no treatment is given. Ursodeoxycholic acid (UDCA) and obeticholic acid (OCA) are the two licensed therapies for PBC in the USA and Europe (1). Nevertheless, the hepatobiliary biochemical data tend to completely normalize in only 20-30% of UDCA-treated PBC patients (3), and up to 60% of UDCA suboptimal responders do not respond to OCA (4). Moreover, among PBC patients with cirrhosis, the response to UDCA is dramatically decreased, and the dose of OCA needs adjustment, as mortality related to OCA usage has been reported in PBC patients with moderate to severe liver function impairment. Early treatment and monitoring of the therapeutic response of PBC might thus save the majority of patients from serious complications.

AMA titers are classically reported to not correlate with disease severity or progression in PBC patients (5). However, AMAs are present in approximately 90% of PBC patients (1), and AMAs usually appear before symptoms of hepatobiliary abnormalities; thus, the production of AMAs is likely not an epiphenomenon in PBC patients (6). Intriguingly, some evidence suggests that AMA titers might be linked with disease severity in PBC patients, given that increasing serum levels of immunoglobulin M (IgM), alkaline phosphatase (alk-p), and γ-glutamyl transferase (γ-GT) and symptoms parallel increasing AMA titers (7), and the presence of immunoglobulin G (IgG)-AMAs seems to be a characteristic of PBC patients with more severe disease (8). On the other hand, how the AMA titer changes in PBC patients who receive UDCA therapy remains poorly understood. For example, AMA levels were decreased in PBC patients who received UDCA therapy during follow-up (9), decreased serum IgG-AMA titers were associated with biochemical response to UDCA treatment in Chinese PBC patients (10), and treatment with UDCA decreased both the levels of IgG-AMAs and pyruvate dehydrogenase in Japanese PBC patients (11). In contrast, in 20 Japanese PBC patients followed for a mean duration of 13.5 years, 5 patients received UDCA therapy, the AMA titers did not significantly change over time regardless of UDCA therapy (12), and UDCA was found to reduce the IgM-AMA but not IgG-AMA levels in peripheral blood mononuclear cells from Japanese PBC patients after bacterial CpG challenge (13). In another study of 19 Japanese PBC patients treated with UDCA, the AMA titers, as determined by immunofluorescence analysis, did not change in 47% of the patients, and the other patients showed increased, decreased, or fluctuating AMA titers during a follow-up >16 months (14). Moreover, a study of 27 USA PBC patients showed, with a median follow-up time of 20 years, IgG-AMA titers did not change significantly over time (15).

In addition to AMA, the autoantibody profile of PBC includes antinuclear antibody (ANA) are noted in up to 50% of PBC patients. Two immunofluorescence patterns of ANAs are considered ‘PBC-specific’: the multiple nuclear dots and rim-like/membranous patterns, and their positivity strongly suggests the diagnosis of PBC, irrespective of AMA status (16). The target antigens of the multiple nuclear dots pattern include sp100, whereas the rim-like/membranous pattern is given by autoantibodies recognizing multiple proteins such as glycoprotein 210 (gp210) (17). Particularly, anti-gp210 antibodies are a strong risk factor for progression to jaundice and hepatic failure (18). However, the specific ANA patterns, as well as sp100 and gp210 autoantibodies were mostly not assessed in early years. Thus, unlike AMA titers, the longitudinal ANA-associated profiles are not available to correlate the clinical outcomes of PBC patients.

Accordingly, we conducted a retrospective study of a 28-year hospital-based cohort to elucidate the precise changes in AMA titers and liver biochemistry levels in 157 PBC patients with different UDCA responses in Taiwan, an Asian country where OCA is not yet available for treating PBC patients.

## Methods

### Patients

Patients older than 18 years old with a diagnosis of PBC were consecutively enrolled at a Taiwan tertiary referral center between July 1988 and August 2020. The diagnosis of PBC was based on the presence of at least 2 of 3 key criteria: (1) an AMA titer higher than 1:40, (2) abnormal alkaline phosphatase (Alk-p) levels [≥1.5 X upper limit of normal (ULN)], and (3) liver histology compatible with PBC. Subjects with autoimmune hepatitis, human immunodeficiency virus, hepatitis B virus (HBV) infection or hepatitis C virus (HCV) infection, hemochromatosis, primary cancer, or alcoholism and recipients of solid organ transplants were excluded.

### Study Design

The clinical details of each patient, including baseline demographic data and biochemistry results, were collected and analyzed. Biochemistry tests, including AMA (Indirect immunofluorescence assays for detecting IgG. Year 1988-2018: Diasorin Molecular LLC, Cypress, CA 90630 (catalog no.1741); year 2018-2020 (catalog no.: FA 1620-2020-1), EUROIMMUN, Lübeck, Germany), ANA (EUROIMMUN), aspartate aminotransferase (AST), alanine aminotransferase (ALT), total bilirubin (Bil (T)), Alk-p, γ-GT, albumin, alpha fetoprotein (AFP), estimated glomerular filtration rate (eGFR), and platelet count tests, were assessed in the serum of the enrolled participants at baseline and every 3-6 months during follow-up. The aforementioned biochemistry values were measured using routine automated techniques in the clinical pathology department of the hospital. The AMA titers assessed by using Diasorin and EUROIMMUN kits had been compared by using the same 50 AMA-positive and 50 AMA-negative patients’ serum, and the titer differences between the two kits were negligible (< ± 2). A diagnosis of liver cirrhosis was made based on early histologic findings or ultrasonographic findings compatible with cirrhosis; the occurrence of esophageal or gastric varices, splenomegaly and/or thrombocytopenia were used as additional signs to make a diagnosis, as described previously (19). Liver ultrasound was performed every 6 months to monitor for cirrhosis and hepatocellular carcinoma (HCC). HCC was diagnosed by histology/cytology or ultrasonographic findings in addition to high AFP levels or typical imaging findings, as described previously (20). All enrolled PBC patients were treated with UDCA at a standard dose of 13-15 mg/kg/day. Patients with Alk-p levels >1.67 × upper limit of normal after 1 year of treatment were defined as nonresponders (21).

### Statistical Analyses

All statistical analyses were performed using the Statistical Package for the Social Sciences (SPSS version 21, SPSS Inc., Chicago, IL, USA) or MedCalc (MedCalc ver. 12.4, MedCalc Software Corp., Acacialaan, Oostend, Belgium) software. Continuous variables are presented as the mean ± standard deviation/median (range), and categorical variables are presented as numbers and percentages (%). For comparisons between groups, continuous variables were analyzed using Student’s t-tests, whereas categorical variables were analyzed using the chi-squared test or Fisher’s exact test as appropriate. Univariate logistic, Kaplan-Meier or univariate Cox regression analyses were used to assess the relationships among various variables and patient events. Multivariate logistic or Cox regression models were used to assess relationships between various dependent and independent variables by considering all the independent variables with *p* values <0.05 in the univariate analyses. Receiver operating characteristic (ROC) curve analyses were performed to evaluate whether independent variables were predictors of dependent variables. The Youden index was used to identify the optimum cutoff values for the independent variables from the coordinate points of the ROC curves. Longitudinal alterations in biochemistry profiles within the same patients were analyzed and compared using analysis of variance, employing repeated-measures general linear models. Paired t-tests or nonparametric methods (Wilcoxon test) were used for the same variable measured in the same subject at >2 time points if Mauchly’s sphericity test yielded *p* values <0.05 during repeated measures. Statistical significance was defined at the 5% level based on two-tailed tests.

### Institutional Review Board

The study was conducted in accordance with good clinical practice and all applicable regulations, including the Declaration of Helsinki and local regulatory requirements, and was approved by the ethics committee of Chang Gung Memorial Hospital. The need for consent was waived because there are minimal risks to subjects in the current study.

## Results

### Baseline Characteristics

A total of 157 consecutive patients with PBC were enrolled in the current study. The baseline characteristics are listed in **Table 1**. At baseline, of 157 patients, 51 (32.5%) had cirrhosis, 128 (81.5%) had detectable AMAs, and 136 (86.6%) were female, with a female-to-male ratio of 6.476:1 and a mean and median age of 54.41 and 54.00 years, respectively.

**Table 1:** Baseline characteristics of the patients with primary biliary cholangitis [mean+/-standard deviation/median (range)].

	All (n = 157)	Female (n = 136)	Male (n = 21)	*p* values (males vs. females)
Age (years)	54.41 ± 11.38/54.00 (25~80)	54.19 ± 11.08/54.0 (25~80)	55.81 ± 13.36/52.0(36~77)	0.546
AMA (diluted titer)*	410.8 ± 474.9/160.0 (0~1280)	387.9 ± 461.4/160.0 (0~1280)	560.0 ± 545.8/320 (0~1280)	0.142
ANA (diluted titer)*	480.7 ± 556.8/160.0 (0~1280)	499.0 ± 558.0/320.0 (0~1280)	335.3 ± 546.4/0 (0~1280)	0.32
AST(U/L)	98.63 ± 74.47/83.5 (14~511)	98.73 ± 74.42/85.0 (14~488)	98.0 ± 102.7/68.0 (15~511)	0.969
ALT(U/L)	99.49 ± 98.43/79.0 (9~978)	94.60 ± 70.80/85.0 (14~488)	130.2 ± 198.9/87 (19~978)	0.124
Alk-p (U/L)	341.1 ± 221.1/302 (57~1341)	343.8 ± 229.5/303 (57~1341)	323.6 ± 160.1/272 (123~670)	0.704
γ-GT (U/L)	328.9 ± 324.1/229 (13~2306)	322 ± 329.9/226(13~2306)	382.6 ± 278.6/306 (100~1065)	0.483
Total bilirubin (mg/dL)	2.66 ± 4.89/1.2 (0.3~46.4)	2.81 ± 5.46/1.1 (0.2~48.6)	2.72 ± 1.74/2.6 (0.5~6.5)	0.941
Albumin (g/dL)	4.03 ± 0.598/4.10 (2~5)	4.0 ± 0.493/4.0 (2~5)	4.23 ± 0.6/4.27 (3~5)	0.127
eGFR (ml/min/1.73m^2^)	89.63 ± 47.67/80.33 (12~179)	93.53 ± 45.57/86.62 (27~179)	89.88 ± 50.23/70.2 (12~101)	0.096
Platelet (K/uL)	206.7 ± 84.8/190.5 (24~403)	207.5 ± 88.8/193.5 (24~403)	202.2 ± 59.9/182.5 (123~301)	0.845
AFP (ng/mL)	3.88 ± 1.96/3.00 (1~14)	3.85 ± 1.87/3.0 (1~14)	4.03 ± 2.45/3.0 (2~13)	0.711
Liver cirrhosis, n (%)	51 (32.5)	44 (32.5)	7 (33.3)	0.929

AMA, antimitochondrial antibody; ANA, antinuclear antibody; AST, aspartate transaminase; ALT, alanine aminotransferase; Alk-p, alkaline phosphatase; γ-GT, gamma-glutamyltransferase. eGFR, estimated glomerular filtration rate; AFP, alpha fetoprotein; *, The diluted titer “1, X” was presented as “X”, and the mean+standard deviation/median (range) of X was shown in the table. For example, the diluted titers such as 1,40, 1,80, 1,160, 1,320, 1,640, 1,1280 were presented as 40, 80, 160, 320, 640 and 1280, respectively.

### Baseline Factors Associated With UDCA Response

Compared with UDCA responders, UDCA nonresponders were younger and had higher levels of Alk-p and Bil (T) and rates of cirrhosis (**Supplementary Table 1**). The univariate analyses showed that baseline age was positively associated with UDCA response and that Alk-p and Bil (T) levels and cirrhosis were negatively associated with UDCA response. The multivariate analysis showed that baseline Alk-p levels and cirrhosis were independently and negatively associated with the UDCA response (**Table 2**).

**Table 2:** Baseline factors associated with UDCA response.

	Univariate analyses		Multivariate analyses	
	OR (95% CI OR)	*p* values	OR (95% OR)	*p* values
Sex (male)	0.848 (0.305~2.361)	0.753		
Age (years)	1.046 (1.012~1.082)	0.008	1.039 (0.995~1.084)	0.083
AMA (diluted titer)*	1.001 (1.00~1.001)	0.14		
ANA (diluted titer)*	1.00 (0.99~1.000)	0.44		
AST(U/L)	0.997 (0.991~1.002)	0.228		
ALT(U/L)	1.00 (0.996~1.003)	0.808		
Alk-p (U/L)	0.993 (0.990~0.996)	<0.001	0.995 (0.992~0.998)	0.002
γ-GT (U/L)	1.00 (0.998~1.001)	0.515		
Total bilirubin (mg/dL)	0.625 (0.446~0.875)	0.006	0.767 (0.568~1.034)	0.082
Albumin (g/dL)	1.798 (0.785~4.119)	0.165		
eGFR (ml/min/1.73m^2^)	0.986 (0.965~1.007)	0.2		
Platelet (K/uL)	1.001 (0.965~1.007)	0.762		
AFP (ng/mL)	1.037 (0.856~1.255)	0.713		
Liver cirrhosis, (yes/no)	0.325 (0.141~0.750)	0.008	0.28 (0.096~0.819)	0.02

UDCA, ursodeoxycholic acid; OR, odds ratio; 95% CI, 95% confidence interval; AMA, antimitochondrial antibody; ANA, antinuclear antibody; AST, aspartate transaminase; ALT, alanine aminotransferase; Alk-p, alkaline phosphatase; γ-GT, gamma-glutamyltransferase. eGFR, estimated glomerular filtration rate; AFP, alpha fetoprotein. *, The diluted titer “1, X” was presented as “X”. For example, the diluted titers such as 1,40, 1,80, 1,160, 1,320, 1,640, 1,1280 were presented as 40, 80, 160, 320, 640 and 1280, respectively.

### Longitudinal Trends of Biochemistry

The follow-up duration was up to 28.73 years (mean+/- standard deviation: 7.50+/-6.68 years; median: 6.42 years; range: 0.5 to 28.73 years). Among the 157 patients, 145 were followed for more than 1 year, the levels of AST, ALT, Alk-p and γ-GT at 1 year after UDCA treatment had decreased from those at baseline; at the last follow-up, the AMA titers and the levels of ALT, Alk-p and γ-GT had decreased (**Table 3**).

**Table 3:** Longitudinal alterations of biochemistry of the PBC patients.

	Baseline levels	Levels after 1 year	Levels at last follow-up	Paired *p* values 1#	Paired *p* values 2*
AMA (diluted titer)**	410.8 ± 474.9	321.6 ± 421.85	218.9 ± 335.9	0.08	<0.001
UDCA response (+)	501.6 ± 526.7	331.3 ± 405.6	162.0 ± 252.6	0.028	<0.001
UDCA response (-)	370.4 ± 423.4	311.8 ± 412.8	219.4 ± 301.5	0.814	0.171
ANA (diluted titer)**	480.7 ± 556.8	535.5 ± 552.1	467.1 ± 534.9	0.865	0.984
UDCA response (+)	385.0 ± 540.6	305.2 ± 478.9	274.3 ± 476.3	0.837	0.191
UDCA response (-)	472.6 ± 531.9	756.9 ± 534.1	598.6 ± 530.1	0.724	0.788
**AST(U/L)**	98.63 ± 74.47	69.48 ± 73.98	89.07 ± 243.54	0.003	0.635
UDCA response (+)	83.8 ± 101.3	39.82 ± 33.93	45.58 ± 38.42	0.003	0.014
UDCA response (-)	101.7 ± 55.5	91.72 ± 81.23	120.0 ± 355.6	0.233	0.669
**ALT(U/L)**	99.49 ± 98.43	63.58 ± 68.20	61.03 ± 88.33	0.002	<0.001
UDCA response (+)	95.10 ± 144.6	39.92 ± 28.61	39.12 ± 30.65	0.009	0.009
UDCA response (-)	99.75 ± 57.58	81.4 ± 82.7	73.0 ± 122.0	0.077	0.088
**Alk-p (U/L)**	341.1 ± 221.1	214.6 ± 138.4	216.8 ± 180.1	<0.001	<0.001
UDCA response (+)	214.4 ± 140.5	123.6 ± 60.8	130.3 ± 60.9	<0.001	<0.001
UDCA response (-)	408.9 ± 218.9	284.1 ± 140.9	229.1 ± 154.7	<0.001	<0.001
**γ-GT (U/L)**	328.9 ± 324.1	177.2 ± 192.8	174.2 ± 177.4	<0.001	<0.001
UDCA response (+)	282.5 ± 387.6	134.3 ± 207.1	120.7 ± 131.6	<0.001	0.007
UDCA response (-)	322.6 ± 245.6	213.1 ± 173.9	170.5 ± 155.5	<0.001	<0.001
**Total bilirubin (mg/dL)**	2.66 ± 4.89	2.37 ± 4.31	4.06 ± 6.78	0.122	<0.001
UDCA response (+)	1.26 ± 1.74	1.03 ± 1.56	2.02 ± 4.62	0.311	0.495
UDCA response (-)	2.54 ± 2.19	3.39 ± 5.35	4.51 ± 6.99	0.052	0.006

PBC, primary biliary cholangitis; AMA, antimitochondrial antibody; ANA, antinuclear antibody; UDCA, ursodeoxycholic acid; AST, aspartate transaminase; ALT, alanine aminotransferase; Alk-p, alkaline phosphatase; γ-GT, gamma-glutamyltransferase. #, paired-t-test p values for the comparisons between levels at baseline and levels after 1 year for variable variables.* paired-t-test p values for comparisons between levels at baseline and levels at final follow-up for variable variables; **, The diluted titer “1, X” was presented as “X”, and the mean ± standard deviation of X was shown in the table. For example, the diluted titers such as 1,40, 1,80, 1,160, 1,320, 1,640, 1,1280 were presented as 40, 80, 160, 320, 640 and 1280, respectively.

### Impact of UDCA Response on Biochemistry Values

**Figure 1:**
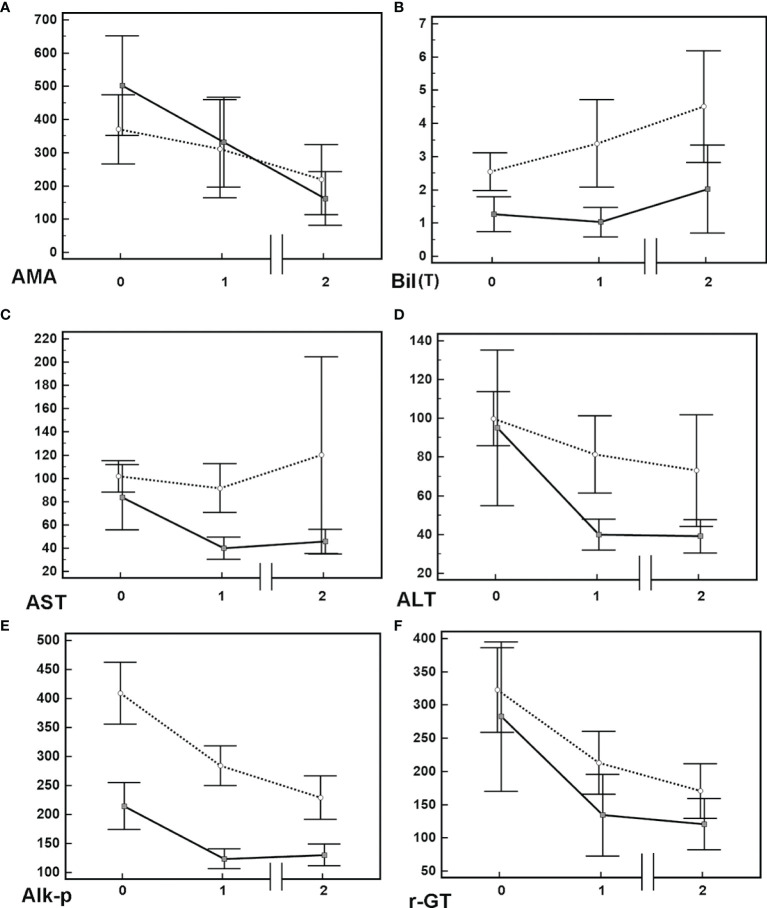
Mean+/-95% confidence interval of various biochemistry levels in PBC patients. **(A)** Anti-mitochondrial Ab [AMA, titrated titers (indirect immunofluorescence assays)], **(B)** total bilirubin [Bil (T) (mg/dL)]. **(C)** aspartate aminotransferase [AST(U/L)]. **(D)** alanine aminotransferase [ALT(U/L)]. **(E)** alkaline phosphatase [Alk-p (U/L)]. **(F)** γ-glutamyltransferase [-GT (U/L)]. Solid lines: data of patients with UDCA response; dashed lines: data of patients without UDCA response. Time 0: baseline; Time 1: 1 year after usage of UDCA; Time 2: final follow-up after usage of UDCA. Solid lines: data of patients with UDCA response; dashed lines: data of patients without UDCA response.

Of the 145 patients who had been followed over 1 year, 84 (57.9%) and 61 (42.1%) were UDCA responders and nonresponders, respectively. When we stratified the patients by the UDCA response, the levels of Alk-p and γ-GT at 1 year after UDCA treatment had decreased compared with those at baseline, and they remained decreased until the end of follow-up in both UDCA responders and nonresponders. However, only UDCA responders had significant decreases in AMA titers and AST and ALT levels, and only UDCA nonresponders exhibited increased Bil (T) levels (**Table 3**, **Figure 1** and **Supplementary Figure 1**). Given that only 128 PBC patients had detectable AMA titers at baseline, we performed subgroup analysis for these 128 patients to determine whether the AMA titer differed according to UDCA response. Only UDCA responders had decreased AMA titers after 1 year of UDCA treatment compared with those at baseline (**Supplementary Table 2**). Of the 128 patients with detectable AMA titers at baseline, vanished levels of AMA were noted in 10 (7.8%) at last follow-up.

### Impact of Baseline Cirrhosis on Biochemistry Values

To determine the effects of liver cirrhosis, we stratified the patients by the presence of baseline cirrhosis. Regardless of baseline cirrhosis, the Alk-p and γ-GT levels were decreased after 1 year of UDCA treatment compared with the baseline levels, and AMA titers were decreased at the end of follow-up. However, only patients with baseline cirrhosis had increased Bil (T) levels at the last follow-up, and only patients without baseline cirrhosis had decreased AST and ALT levels after 1 year of UDCA treatment (**Table 4**).

**Table 4:** Longitudinal alterations of biochemistry of the patients with and without baseline cirrhosis.

	Baseline levels	Levels after 1 year	Levels at last follow-up	Paired *p* values 1#	Paired *p* values 2*
AMA (diluted titer)**					
Baseline cirrhosis (+)	482.2 ± 491.7	400.0 ± 462.4	196.0 ± 322.2	0.389	0.008
Baseline cirrhosis (-)	390.9 ± 461.8	305.9 ± 413.7	201.7 ± 302.6	0.132	0.002
ANA (diluted titer)**					
Baseline cirrhosis (+)	312.7 ± 401.9	487.2 ± 461.5	602.6 ± 597.7	0.159	0.634
Baseline cirrhosis (-)	614.1 ± 587.3	576.1 ± 615.3	485.8 ± 539.8	0.709	0.628
**AST(U/L)**					
Baseline cirrhosis (+)	102.5 ± 68.0	95.3 ± 89.4	151.1 ± 423.0	0.625	0.417
Baseline cirrhosis (-)	91.5 ± 84.5	56.8 ± 63.4	58.9 ± 43.7	0.001	<0.001
**ALT(U/L)**					
Baseline cirrhosis (+)	77.9 ± 42.1	73.6 ± 59.2	79.5 ± 145.4	0.655	0.858
Baseline cirrhosis (-)	107.3 ± 122.5	58.0 ± 72.2	50.7 ± 36.8	0.001	<0.001
**Alk-p (U/L)**					
Baseline cirrhosis (+)	354.6 ± 188.7	262.6 ± 130.4	239.2 ± 178.3	0.001	<0.001
Baseline cirrhosis (-)	315.6 ± 224.1	192.7 ± 137.5	205.5 ± 178.5	<0.001	<0.001
**γ-GT (U/L)**					
Baseline cirrhosis (+)	349.5 ± 419.1	212.65 ± 262.160	120.87 ± 131.412	0.001	0.002
Baseline cirrhosis (-)	287.4 ± 260.9	148.83 ± 134.393	157.58 ± 146.182	<0.001	<0.001
**Total bilirubin (mg/dL)**					
Baseline cirrhosis (+)	2.78 ± 2.56	4.45 ± 6.76	6.84 ± 9.00	0.111	0.024
Baseline cirrhosis (-)	1.56 ± 1.70	1.60 ± 2.49	1.88 ± 3.30	0.879	0.313

AMA, antimitochondrial antibody; ANA, antinuclear antibody; AST, aspartate transaminase; ALT, alanine aminotransferase; Alk-p, alkaline phosphatase; γ-GT, gamma-glutamyltransferase. #, paired-t-test p values for the comparisons between levels at baseline and levels after 1 year for variable variables.* paired-t-test p values for comparisons between levels at baseline and levels at final follow-up for variable variables; **, The diluted titer “1, X” was presented as “X”, and the mean ± standard deviation of X was shown in the table. For example, the diluted titers such as 1,40, 1,80, 1,160, 1,320, 1,640, 1,1280 were presented as 40, 80, 160, 320, 640 and 1280, respectively.

### AMA Titer and HCC

Of the 157 patients, 7 (4.45%) developed HCC during the follow-up. The 28-year cumulative incidence of HCC was 11.3%. The univariate analyses showed that baseline age and AMA titer were associated with the development of HCC. Multivariate analysis confirmed that baseline age was independently associated with HCC risk [Hazard ratio (HR):1.143; 95% confidence interval (CI) of HR:1.051~1.243] (**Supplementary Table 3**). The cutoff value for age as a predictor of developing HCC was 63 years (95% CI of ROC curve: 0.713%~0.847%; Youden J index: 0.6571, sensitivity: 85.71%, specificity: 80.00%).

### AMA Titer and Liver Cirrhosis

Of the 157 patients, 83 (52.9%) developed cirrhosis during follow-up. The cumulative incidence of liver cirrhosis was 66.7%. The univariate analyses showed that baseline age, AMA titer, platelet count, Bil (T) and albumin levels, and UDCA response were associated with liver cirrhosis development. The multivariate analysis confirmed that baseline Bil (T) level (HR: 2.349; 95% CI HR: 1.559~3.541) and platelet count (HR: 0.994; 95% CI HR:0.985~0.997) were independently associated with cirrhosis development (**Supplementary Table 4**).

## Discussion

The most compelling results of the current study are as follows: (1) At baseline, UDCA nonresponders were younger and had higher Alk-p and Bil (T) levels and cirrhosis rates than the UDCA responders. Both baseline Alk-p levels and cirrhosis were independent factors for UDCA response. (2) Regardless of baseline cirrhosis and UDCA response, most PBC patients had decreased Alk-p and γ-GT levels after 1 year of UDCA treatment compared with those at baseline. Patients with baseline cirrhosis or those without UDCA response had increased Bil (T) levels, and patients without baseline cirrhosis or UDCA responders had decreased AST and ALT levels. (3) UDCA responders had a decreased AMA titer at 1 year after UDCA treatment that lasted until the last follow-up. (4) Baseline age was independently associated with HCC development (cutoff age: 63 years). Baseline Bil (T) levels and platelet count were independently associated with cirrhosis development.

The findings that UDCA nonresponders were younger and had higher levels of Alk-p and Bil (T) and cirrhosis rates than UDCA responders and that both baseline cirrhosis and Alk-p levels were independently and negatively associated with UDCA response are consistent with several published ideas: pretreatment parameters, including higher Alk-p and Bil (T) levels and younger age, are associated with a lower likelihood to respond to UDCA (22), younger age at onset and advanced disease at presentation are baseline predictors of poor outcome (21), and histological presence of cirrhosis is associated with progressive disease and poor outcome in PBC patients. All of these findings support the reliability of the results of the current study. Unexpectedly, most PBC patients had decreased γ-GT and Alk-p levels, which is a marker of UDCA response (21), after 1 year of UDCA treatment, regardless of baseline cirrhosis and UDCA response. The decreases in Alk-p and γ-GT levels in PBC patients highlight the efficacy of UDCA in improving cholestasis features, and the efficacy of UDCA has been documented to be superior to that of placebo in a multicenter, double-blind, placebo-controlled trial (23). This result suggests that UDCA treatment should be continued in all PBC patients regardless of baseline cirrhosis and UDCA response, particularly in Taiwan, where the 2nd-line therapy for PBC, OCA, is not yet available. However, deterioration of hepatobiliary function seems to be unpreventable in PBC patients with baseline cirrhosis and UDCA nonresponders, as evidenced by the increased bilirubin levels and the persistently high aminotransaminase levels.

Of note, this 28-year cohort study showed that only UDCA responders had decreased AMA (IgG) titers at 1 year after UDCA treatment that persisted until the last follow-up. Interestingly, the triad of PBC monocytes, biliary apoptosis and AMAs leads to an intense proinflammatory cytokine burst (24). Although the precise pathogenesis of PBC remains unclear, it has been postulated that B cells are involved in the ongoing inflammatory process, as B cell depletion with rituximab significantly reduced the number of AMA-producing B cells, AMA titers, and Alk-p levels (25). Moreover, among AMA-positive patients without PBC, mortality is increased irrespective of the risk of PBC development (26). Among AMA-positive patients with normal cholestatic liver enzymes, those with nonspecific histological findings have lower titers of AMAs than those with liver histology compatible with early-stage PBC (27). Additionally, autoantibody profile was a quantitatively and qualitatively more robust factor in definite PBC than in AMA-positive biochemically normal individuals (28), while increased IgG- and IgA-AMA titers during follow-up were associated with biochemically and/or histologically advanced disease in PBC patients (29). On the other hand, a decreased AMA titer was shown in approximately half of asymptomatic PBC patients treated with fenofibrate (30). There is a strong association between AMAs and an increased serum IgM level (31). Furthermore, the serum titer of AMAs is significantly higher in younger patients with PBC than in older patients with PBC (12), and young age at onset indicates poor prognosis for PBC, as mentioned above (21). Additionally, a low AMA titer may be transient and may occur in many patients without PBC (32), and up to 40.6% of patients with acute liver failure had transient positive AMA (33). All of the above results support that the AMA titer might be linked, either directly or indirectly, with the outcomes of PBC patients, although such links have not been proven statistically, especially in small-scale studies with inherent bias from host heterogeneity. The presence of IgG-AMAs seems to be a characteristic of PBC patients with more severe disease (8). In particular, AMAs are not restricted to a specific IgG subclass, and AMAs of the IgG3 subclass are associated with a more severe disease course (34). Moreover, it has been reported that the AMA titer is not influenced by the immunogenicity of M2 proteins but by the number of M2 proteins that elicit an antibody response (35). Thus, the inconclusive data regarding AMA titers and PBC disease severity might at least partly result from the heterogeneity of AMAs. As mentioned above, decreased titers of serum IgG-AMAs have been reported to be associated with biochemical response to UDCA treatment in Chinese PBC patients (10). Interestingly, most studies conducted in Japan (12–14) have shown that UDCA response does not lead to a decreased AMA titer. Moreover, the serum titer of AMAs might be related to the genotype of glutathione S-transferase (35). Whether genetic or ethnic variations account for the discrepancy in AMA titer changes in UDCA responders demands further evaluation.

Unlike the elevated risk associated with chronic viral (HBV or HCV) infections, it has been reported that HCC is less common in liver cirrhosis caused by autoimmune liver diseases (36) such as PBC. Consistently, the 16-year HCC cumulative incidence in chronic HBV-infected patients is 16.8% (16), being higher than the 28-year HCC cumulative incidence (11.3%) of the current study. Regulatory T cells (Tregs) are the most abundant suppressive cells in the tumor microenvironment and their presence has been correlated with tumor progression. Chronic viral infections are immunologically characterized by a host immune response suppression driven by Tregs (37), by contrast, autoimmune liver diseases are related to functional defect of Tregs (38). The different autoimmune inflammatory activity regulations in viral infections and autoimmune liver diseases might account for the different HCC incidences. Besides, in the current cohort, baseline age was the only independent factor for HCC development after adjusting for baseline AMA titer, which was positively associated with HCC development in the univariate analysis. In addition, although the positive association of baseline AMA titer with HCC development was abolished when considering baseline age, that a high baseline AMA titer was linked with HCC risk supports the idea that AMA titer is associated with PBC disease severity. Likewise, the association between AMA titer and cirrhosis development, albeit not an independent association, suggests that AMA titer might be linked to the prognosis of PBC patients.

The current study has several limitations. First, because of the retrospective nature of the study, a thorough assessment regarding the immunological basis of the altered AMA titers could not be performed. Second, although a negligible drug bias was suggested, as all the enrolled patients were prescribed UDCA at a dose of 13-15 mg/kg/day according to chart records, precise drug compliance of individual patients cannot be guaranteed. Third, since a liver biopsy was not performed for most patients to confirm liver cirrhosis, the diagnosis of cirrhosis might have not been optimal in some patients. Fourth, PBC-specific antinuclear antibodies (ANAs) including anti-sp100 and anti-gp210 (1), or antibodies to kelch-like 12 (KLHL12) and hexokinase 1 (HK-1) (39) might aid to diagnosis the AMA-negative PBC, correlate the prognosis and AMA-associated results of PBC patients. However, these autoantibodies were not assessed in most labs in Taiwan, and the associated data thus are not available in in the current study. Future prospective, randomized controlled studies of PBC patients with histologically proven liver cirrhosis treated with uniform UDCA or OCA regimens are required to verify the association between decreased AMA titers and therapeutic response in patients.

Taken together, the findings of this 28-year cohort study show that most PBC patients treated with UDCA exhibit improved cholestatic features. Only UDCA responders exhibited improvements in hepatobiliary injury, and UDCA nonresponders had increased bilirubin levels. Moreover, UDCA responders had decreased IgG-AMA titers after 1 year of UDCA treatment. These findings highlight the importance of monitoring PBC patients treated with UDCA from a clinical perspective and present AMAs as a potential immunology-based therapeutic target for PBC.

## Data Availability Statement

The raw data supporting the conclusions of this article will be made available by the authors, without undue reservation.

## Ethics Statement

The studies involving human participants were reviewed and approved by Chang Gung Memorial Hospital (IRB No:104-7005B). The need for consent was waived because there are minimal risks to subjects in the current study.

## Author Contributions

M-LC and R-NC, study design and implementation, manuscript drafting, and critical revision of the manuscript for important intellectual content. C-YL, T-MC, W-TC, M-YC and S-CC, data collection and manuscript writing. All authors contributed to the article and approved the submitted version.

## Funding

This study was supported by grants from the Chang Gung Medical Research Program (CMRPG3I0413, CMRPG3L1191, CMRPG3M0211 and CMRPG1K0111-3), and the National Science Council, Taiwan (MOST 110-2629-B-182-001- and 110-2314-B-182-044-). The funders had no role in study design, data collection and analysis, decision to publish, or preparation of the manuscript. The opinions expressed in this paper are those of the authors and do not necessarily represent those of Chang Gung Medical Hospital and National Science Council, Taiwan.

## Conflict of Interest

The authors declare that the research was conducted in the absence of any commercial or financial relationships that could be construed as a potential conflict of interest.

## Publisher’s Note

All claims expressed in this article are solely those of the authors and do not necessarily represent those of their affiliated organizations, or those of the publisher, the editors and the reviewers. Any product that may be evaluated in this article, or claim that may be made by its manufacturer, is not guaranteed or endorsed by the publisher.

## Supplementary Material

Supplementary Figure 1 Scatter plots for longitudinal alterations in various biochemistry parameters in PBC patients. **(A)** Anti-mitochondrial Ab (AMA, titrated titers [indirect immunofluorescence assays)], **(B)** total bilirubin [Bil (T) (mg/dL)]. **(C)** aspartate aminotransferase [AST(U/L)]. **(D)** alanine aminotransferase [ALT(U/L)]. **(E)** alkaline phosphatase [Alk-p (U/L)]. **(F)** γ-glutamyltransferase [γ-GT (U/L)]. Blue lines: data of patients with UDCA response; red lines: data of patients without UDCA response.
