# Supplementary figures and images for: Anti-Mitochondrial Antibody Titers Decrease Over Time in Primary Biliary Cholangitis Patients With Ursodeoxycholic Acid Therapeutic Response: A Cohort Study Followed Up to 28 Years
Source: Front Immunol. 2022 May 19;13:869018. doi: 10.3389/fimmu.2022.869018 (PMC9160714; doi:10.3389/fimmu.2022.869018)

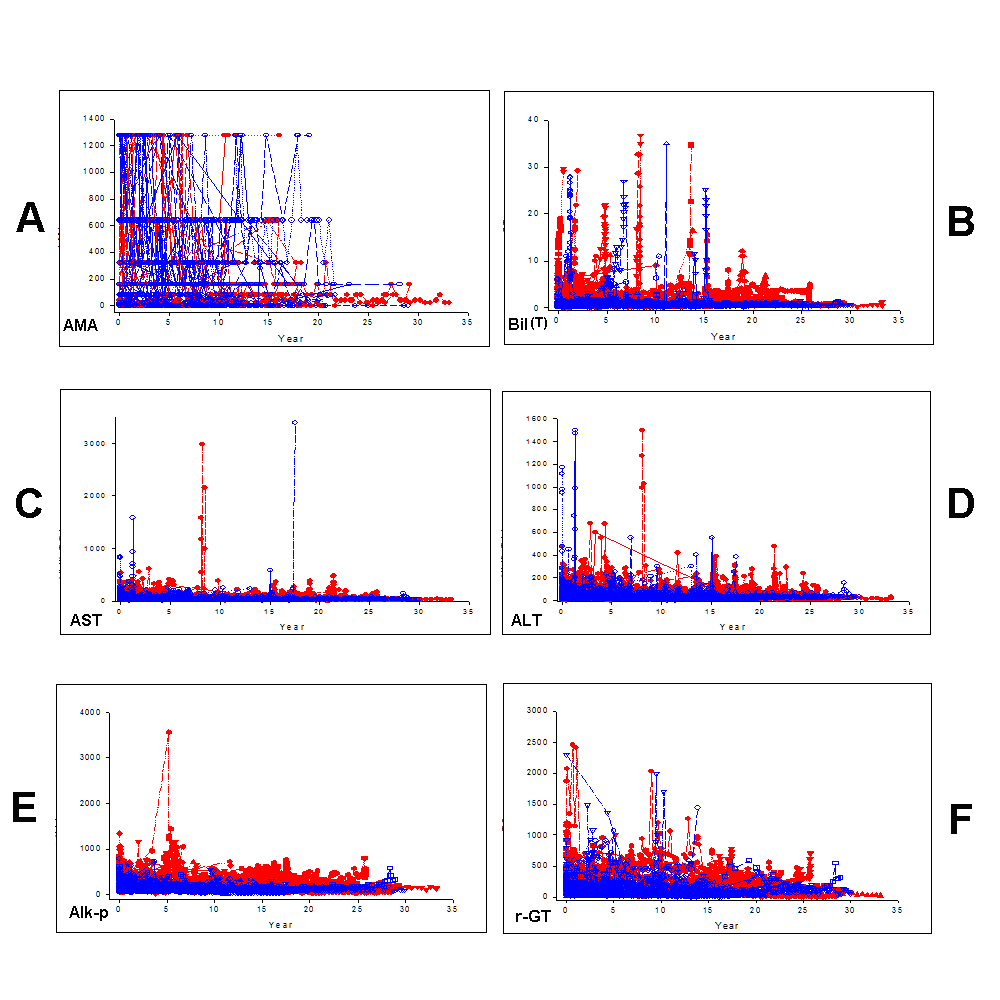

Supplement: Supplementary Figure 1 — Scatter plots for longitudinal alterations in various biochemistry parameters in PBC patients. (A) Anti-mitochondrial Ab (AMA, titrated titers [indirect immunofluorescence assays)], (B) total bilirubin [Bil (T) (mg/dL)]. (C) aspartate aminotransferase [AST(U/L)]. (D) alanine aminotransferase [ALT(U/L)]. (E) alkaline phosphatase [Alk-p (U/L)]. (F) γ-glutamyltransferase [γ-GT (U/L)]. Blue lines: data of patients with UDCA response; red lines: data of patients without UDCA response. [file Image_1.tif]
