# Supplementary material for: Anti-Mitochondrial Antibody Titers Decrease Over Time in Primary Biliary Cholangitis Patients With Ursodeoxycholic Acid Therapeutic Response: A Cohort Study Followed Up to 28 Years
Source: Front Immunol. 2022 May 19;13:869018. doi: 10.3389/fimmu.2022.869018 (PMC9160714; doi:10.3389/fimmu.2022.869018)
Supplement: Supplementary file 2 [file Table_1.doc]

**Supplementary Table 1. Comparisons between UDCA responder and non-responders**

**[mean+/-standard deviation/median (range)]**.

|  | UDCA responder (n=84) | UDCA non-responder (n=61) | *p* values |
| --- | --- | --- | --- |
| Sex (male), n (%) | 12 (14.3) | 9 (15.0) | 0.755 |
| Age (yrs) | 57.21±10.67/57.50 (34~80) | 51.44±11.80/50.0(25~74) | 0.006 |
| AMA (diluted titer)** | 501.6±526.7/320.0 (0~1280) | 370.4±423.4/240 (0~1280) | 0.153 |
| ANA (diluted titer)** | 385.0±540.6/40.0 (0~1280) | 472.6±531.9/320 (0~1280) | 0.445 |
| AST(U/L) | 83.8±101.3/57.0 (15~511) | 101.7±55.5/90.0 (17~310) | 0.213 |
| ALT(U/L) | 95.10±144.6/62.5 (9~978) | 99.75±57.58/96.0 (19~289) | 0.809 |
| Alk-p (U/L) | 214.4±140.5/166.0 (66~681) | 408.9±218.9/368.0 (65~1291) | <0.001 |
| r-GT (U/L) | 282.5±387.6/166.5 (13~2306) | 322.6±245.6/270.0 (13~1148) | 0.514 |
| Total bilirubin (mg/dL) | 1.26±1.74/0.9 (0.4~11.0) | 2.54±2.19/1.8 (0.5~10.3) | 0.001 |
| Albumin (g/dL) | 4.17±0.445/4.3 (3~5) | 3.97±0.672/4.10 (2~5) | 0.161 |
| eGFR (ml/min/1.73m^2^) | 74.46±45.26/75.1 (12~179) | 107.37±56.53//100.26 (20~179) | 0.202 |
| Platelet (K/uL) | 213.3±94.1/236.0 (24~349) | 206.5±86.3/189.0 (58~403) | 0.766 |
| AFP (ng/mL) | 4.02±1.86/3.35 (1~13) | 3.87±2.2/3.0 (1~14) | 0.716 |
| Liver cirrhosis, n (%) | 16 (19.0) | 26 (42.6) | 0.005 |

AMA: antimitochondrial antibody; ANA: antinuclear antibody; AST: aspartate transaminase; ALT: alanine aminotransferase; Alk-p: alkaline phosphatase; r-GT: gamma-glutamyltransferase. eGFR: estimated glomerular filtration rate; AFP: alpha fetoprotein; **: The diluted titer “1: X” was presented as “X”, and the mean+standard deviation/median (range) of X was shown in the table.

**Supplementary Table 2. Longitudinal alterations of AMA titer of the patients (baseline AMA+).**

|  | Baseline levels | Levels after 1 year | Levels at last follow-up | Paired *p* values 1# | Paired *p* values 2* |
| --- | --- | --- | --- | --- | --- |
| **AMA (diluted titer)**** |  |  |  |  |  |
| UDCA response (+) | 609.6±527.5 | 408.2±424.4 | 186.0±266.5 | 0.017 | <0.001 |
| UDCA response (-) | 465.7±381.7 | 443.8±470.6 | 296.6±324.4 | 0.815 | 0.128 |

AMA: antimitochondrial antibody; **: 1: The diluted titer “1: X” was presented as “X” and the mean+standard deviation of X was shown in the table.

**Supplementary Table 3. Baseline factors associated with HCC development.**

|  | Univariate analyses | | Multivariate analyses | |
| --- | --- | --- | --- | --- |
|  | HR (95% CI HR) | *p* values | HR (95% CI HR) | *p* values |
| Sex (male) | 0.04 (0.0 ~778.0) | 0.524 |  |  |
| Age (years) | 1.142 (1.058~1.233) | 0.001 | 1.143 (1.051~1.243) | 0.002 |
| AMA (diluted titer) | 1.002 (1.000~1.003) | 0.034 | 1.001 (1.000~1.003) | 0.067 |
| ANA (diluted titer) | 1.001 (1.000~1.003) | 0.077 |  |  |
| AST(U/L) | 0.995 (0.979~1.01) | 0.498 |  |  |
| ALT(U/L) | 0.996 (0.983~1.008) | 0.475 |  |  |
| Alk-p (U/L) | 0.999 (0.994~1.003) | 0.617 |  |  |
| r-GT (U/L) | 1.001 (0.999~1.002) | 0.37 |  |  |
| Total bilirubin (mg/dL) | 1.074 (0.779 ~1.481) | 0.663 |  |  |
| Albumin (g/dL) | 1.098 (0.236~5.095) | 0.905 |  |  |
| eGFR (ml/min/1.73m^2^) | 1.003 (0.96~1.049) | 0.887 |  |  |
| Platelet (K/uL) | 0.989 (0.978~1.000) | 0.053 |  |  |
| AFP (ng/mL) | 0.938 (0.639 ~1.624) | 0.938 |  |  |
| Liver cirrhosis, (yes/no) | 0.439 (0.053 ~3.655) | 0.446 |  |  |
| UDCA response | 4.726 (0.527~42.425) | 0.165 |  |  |

HCC: hepatocellular carcinoma; HR: hazard ratio; 95% CI: 95% confidence interval; AMA: antimitochondrial antibody; ANA: antinuclear antibody; AST: aspartate transaminase; ALT: alanine aminotransferase; Alk-p: alkaline phosphatase; r-GT: gamma-glutamyltransferase. eGFR: estimated glomerular filtration rate; AFP: alpha fetoprotein; UDCA: ursodeoxycholic acid.

**Supplementary Table 4. Baseline factors associated with cirrhosis development.**

|  | Univariate analyses | | Multivariate analyses | |
| --- | --- | --- | --- | --- |
|  | HR (95% CI HR) | *p* values | HR (95% CI HR) | *p* values |
| Sex (male) | 1.123 (0.609 ~2.071) | 0.71 |  |  |
| Age (years) | 1.024 (1.004~1.043) | 0.016 | 1.010 (0.968~1.054) | 0.645 |
| AMA (diluted titer) | 1.000 (1.000~1.001) | 0.047 | 1.001 (1.000~1.002) | 0.117 |
| ANA (diluted titer) | 1.000 (0.999~1.000) | 0.52 |  |  |
| AST(U/L) | 1.001 (0.998~1.003) | 0.462 |  |  |
| ALT(U/L) | 0.998 (0.995~1.001) | 0.188 |  |  |
| Alk-p (U/L) | 1.001 (1.000~1.002) | 0.06 |  |  |
| r-GT (U/L) | 1.000 (0.999~1.001) | 0.987 |  |  |
| Total bilirubin (mg/dL) | 1.087 (1.032~1.146) | 0.002 | 2.349 (1.559~3.541) | <0.001 |
| Albumin (g/dL) | 0.467 (0.312 ~0.698) | <0.001 | 1.075 (0.163~1.409) | 0.863 |
| eGFR (ml/min/1.73m^2^) | 1.008 (0.994~1.002) | 0.271 |  |  |
| Platelet (K/uL) | 0.995 (0.992~0.997) | 0.009 | 0.994 (0.985~0.997) | 0.001 |
| AFP (ng/mL) | 1.004 (0.888 ~1.134) | 0.951 |  |  |
| UDCA response | 0.579 (0.343~0.976) | 0.04 | 0.665 (0.219~2.019) | 0.471 |

HR: hazard ratio; 95% CI: 95% confidence interval; AMA: antimitochondrial antibody; ANA: antinuclear antibody; AST: aspartate transaminase; ALT: alanine aminotransferase; Alk-p: alkaline phosphatase; r-GT: gamma-glutamyltransferase. eGFR: estimated glomerular filtration rate; AFP: alpha fetoprotein; UDCA: ursodeoxycholic acid.
